# Assessing the impact of remote work during COVID-19 on clinical and translational scientists and staff in Colorado

**DOI:** 10.1017/cts.2020.570

**Published:** 2020-12-21

**Authors:** Heather M. Gilmartin, Brigid Connelly, Annika Hebbe, Catherine Battaglia, Bethany M. Kwan

**Affiliations:** 1 Denver/Seattle Center of Innovation for Veteran-Centered and Value Driven Care, VHA Eastern Colorado Healthcare System, Aurora, CO, USA; 2 Health Systems, Management and Policy, School of Public Health, University of Colorado, Aurora, CO, USA; 3 Colorado Clinical & Translational Sciences Institute, Aurora, CO, USA; 4 Department of Family Medicine, University of Colorado Anschutz Medical Campus, Aurora, CO, USA

**Keywords:** Remote work, COVID-19, clinical and translational science

## Abstract

The COVID-19 pandemic has required many clinical and translational scientists and staff to work remotely to prevent the spread of the virus. To understand the impact on research programs, we assessed barriers to remote work and strategies implemented to support virtual engagement and productivity. A mixed-methods RedCap survey querying the remote work experience was emailed to Colorado Clinical and Translational Sciences Institute (CCTSI) scientists and staff in April 2020. Descriptive analyses, Fisher’s Exact tests, and content analysis were conducted. Respondents (*n* = 322) were primarily female (*n* = 240; 75%), 21–73 years old (mean = 42 years) with a PhD (*n* = 139; 44%) or MD (*n* = 56; 55%). Prior to COVID-19, 77% (*n* = 246) never or rarely (0–1 day a week) worked remotely. Remote work somewhat or greatly interfered with 76% (*n* = 244) of researchers’ programs and 71% (*n* = 231) reported slowing or stopping their research. Common barriers included missing interactions with colleagues (*n* = 198; 62%) and the absence of routines (*n* = 137; 43%). Strategies included videoconferencing (*n* = 283; 88%), altering timelines and expectations (*n* = 180; 56%). Scientists and staff experienced interference with their research when they shifted to remote work, causing many to slow or stop research programs. Methods to enhance communication and relationships, support productivity, and collectively cope during remote work are available.

## Introduction

The current novel coronavirus disease 2019 (COVID-19) pandemic has led to substantial changes in society [1]. In March 2020, tens of millions of American workers were instructed to work from home to prevent the spread of the virus that causes COVID-19 (i.e. SARS-COV-2) [2]. Even before local public health orders were made official [3], University campuses and healthcare systems closed academic offices and laboratories, and directed scientists and staff to continue their work remotely where possible. Little was known about how this shift to remote work might impact the scientific community [4].

Early guidance from scientific colleagues was to prioritize work that could be done remotely (e.g. data analysis, writing grants and manuscripts) [5]. In March, it was not imagined that remote work would become a long-term solution to the mitigation of the COVID-19 pandemic. Eight months later, campuses have only partially reopened. Many scientists and researchers have been told to continue remote work for the foreseeable future. There is an urgent need for clinical and translational scientists and staff to learn and share remote work best practices, along with the creation of systematic interventions by University, hospital, and department leadership. It is vital that scientists and staff remain engaged, productive and healthy during the COVID-19 work from home period [4].

Traditionally, research is conducted in-person, within offices, clinical settings, communities, and laboratories. Research is a collaborative work process between scientists, staff, leadership, and participants of research [6]. The laboratory or office setting provides equipment (e.g. microscopes, computers), data sources (e.g. animals, cell lines, patients), and physical resources (e.g. desks, computers, printers, meeting spaces). The daily in-person interactions support social connection, physical closeness, and accountability [6]. Scientists and staff working remotely during COVID-19 are unable to engage with laboratory animals, cell lines, patients, or the community and can be disconnected from one’s team. This can delay vital scientific research [5], decrease research productivity, increase stress, disengagement, burnout and result in turnover intentions of staff [7,8]. Remote work may also disrupt the planning and execution of future research projects that support patient health and safety and may negatively impact the professional development of junior researchers and challenge recruitment and retention of talented research staff [5,8]. The challenge of remote work is further exacerbated by working within the same space as roommates, partners, and children. The intersection of work, children, and home life can be overwhelming, especially for female scientists who bear the burden for the majority of child and home responsibilities [9,10].

At this time, remote work has become a long-term strategy for scientists and staffs as we all continue to strive to control the spread of COVID-19 across the US. The purpose of this study was to systematically collect, analyze, and share remote work experiences, barriers, and strategic workarounds created by clinical and translational scientists and staff in the wake of stay-at-home orders due to the COVID-19 pandemic. Our goal is to share methods created to enhance communication, maintain relationships, and collectively cope during COVID-19 remote work to ensure the short- and long-term success of the clinical and translational science endeavor.

## Methods

### Design and Setting

This study is a cross-sectional, convenience sample design conducted in the Colorado Clinical and Translational Sciences Institute (CCTSI). The CCTSI is a Clinical Translational Sciences Award site funded by the National Center for Advancing Translational Sciences to provide resources to support basic, translational, and clinical researchers to move scientific discoveries to clinical innovations that diagnose, prevent, or treat disease. Based at the University of Colorado Anschutz Medical Campus (UC-AMC), the CCTSI partners with multiple institutions including Children’s Hospital of Colorado, University of Colorado Hospital, Denver Health, Kaiser Foundation Research Institute, National Jewish Hospital, The Rocky Mountain Regional Veterans Health Administration Medical Center, Colorado State University, and the University of Colorado system. All CCTSI partner institutions were required to follow the State of Colorado stay-at-home orders initiated in March 2020. The study was deemed non-human subjects research by the Colorado Multiple Institutional Review Board (20-0892).

### Participants and Recruitment

Survey invitations were sent to current CCTSI members over the age of 18 years old (*n* = 5,067). CCTSI membership is free for University faculty, fellows, residents, students, clinician and non-clinical investigators, and research staff at partner institutions. Membership is required to gain access to the education, training, networking, and grant opportunities provided throughout the year. Information about the study was posted on the CCTSI website and was announced on April 27, 2020 in the UC-AMC Dean’s Newsletter. Over the following 2 weeks, four invitations to participate with a RedCap [11] survey link were emailed to CCTSI members. The study was further promoted via campus newsletters and on Twitter. The CCTSI homepage included an overview of the survey, definitions, contact information for the lead investigator, and the survey link.

### Remote Work Survey

We developed a survey [12] informed by the remote work literature [13–15], experiences posted to Twitter (#remote work; #WFH) in the early days of remote work during COVID-19, and the authors’ personal experiences with remote work. The survey was pilot tested by members of the Denver/Seattle Veterans Health Administration Center of Innovation works-in-progress meeting for ease of understanding and clarity. The following demographics were captured in the survey: Respondent’s age, professional credentials, gender, research role, faculty investigator level, CCTSI partner site, and stage of their research on the clinical and translational research spectrum [16]. Previous remote work experiences were queried using an open text item. Participants rated the extent to which remote work during COVID-19 interfered with their research activities (i.e. does not interfere, interferes somewhat, interferes to a great extent), and selected from a list of common barriers to remote work, and the frequency of these barriers. Open text items were available to report additional barriers and workarounds created to address barriers to remote work.

Respondents were asked if they would be stopping any research during COVID-19 (i.e. none, some, all, not applicable) and were given an option to describe the research put on hold and why. Respondents were asked to select from a list of strategies being implemented by department level leadership, investigators, project leads, or project managers to engage staff in a productive way. Finally, to identify social support in the home, respondents were asked to indicate who else is in their home during the day and/or night.

## Statistical Analyses

Survey data were exported from RedCap to SPSS (IBM, version 27) for descriptive analyses of the quantitative data and to Microsoft Excel v.16.34 (Microsoft Corp) for the qualitative data. The data were stratified by the extent remote work during COVID-19 interfered with research activities. A Fisher’s Exact test was performed to examine the relationship between level of reported interference with research activities due to COVID-19 remote work, demographic variables, and stopping of research using R version 3.5.3. Additionally, a Fisher’s Exact test was performed to assess the relationship between previous remote workdays and demographic variables.

Qualitative responses were analyzed using manifest content analysis [17]. A structured matrix was developed to code the data based on the survey questions. All the text responses were reviewed for content and correspondence for the following questions: other barriers to remote work, reasons for stopping research, and workarounds. Codes and categories were discussed within the analyst team. Face validity of the categorized results were established by the principal investigator (HG). Quotes were used to enhance the credibility of the findings and contextualize the quantitative survey results.

## Results

Of the 5,098 current CCTSI members, 322 responded to the survey. Staff from UC-AMC (*n* = 254; 79%), Children’s Hospital of Colorado (*n* = 87; 27%), University of Colorado Hospital (*n* = 29; 9%); University of Colorado, Boulder (*n* = 20; 6%) and Denver (*n* = 16, 5%), Colorado State University (*n* = 16; 5%), and other CCTSI partner sites participated (Table 1). A majority of respondents were female (*n* = 240; 75%), with ages ranging from 21 to 73 (mean = 42 years). The most common highest educational degree was a PhD (*n* = 139; 44%) or MD (*n* = 56; 55%), followed by a master’s degree (*n* = 79; 25%). Respondents represented a wide range of healthcare professions, including physicians (*n* = 56; 55%), public health professionals (*n* = 25; 25%) nurses, and advanced practice nurses (*n* = 10; 10%). The most frequent research role reported was faculty investigators (*n* = 165; 51%) at the Assistant Professor level (*n* = 67; 41%), followed by research clinical staff (*n* = 105; 33%). Regarding the clinical and translational research spectrum, participants research programs conducted Translation to Practice (*n* = 74, 24%), Translation to Patients (*n* = 48; 15%), or the state of research on the translational research continuum was not known (*n* = 85; 28%) (Table 1).

**Table 1.** Summary of survey responses (*n* = 322)

Questions & Responses		
**What is your age?** Mean (median)	42.2 (40)	
	**N**	**%**
**Educational Degree** (*n* = 318)		
*Associates*	1	0.3
*Bachelors*	46	14.5
*Masters*	79	24.8
*PhD*	139	43.7
*Practice Doctorate (MD, PharmD, JD)*	53	16.7
**Professional Degree** (*n* = 101)		
*Registered Nurse or Advanced Practice Nurse*	10	9.9
*Medical Doctor/Doctor of Osteopathy*	59	58.4
*Dietician/Social Worker/Pharmacist*	7	10
*Public Health*	25	24.8
**Gender** (*n* = 322)		
*Male*	79	24.5
*Female*	240	74.5
*Non-binary*	1	0.3
*Prefer not to answer*	2	0.6
**Research Role** (*n* = 321)		
*Faculty investigator*	165	51.4
*Non-faculty investigator*	24	7.5
*Research support staff*	5	1.6
*Research administration*	22	6.9
*Research clinical staff*	105	32.6
**Faculty Investigator Role** (*n* = 164)		
*Instructor/Senior Instructor*	14	8.5
*Assistant Professor*	67	40.9
*Associate Professor*	42	25.6
*Professor*	38	23.2
*Other*	3	1.7
**CCTSI Partner Site** (*n* = 322)		
*Children’s Hospital Colorado*	87	27
*Denver Health*	6	1.9
*Kaiser Foundation Research Institute*	5	1.6
*National Jewish Hospital*	3	0.9
*University of Colorado Hospital*	29	9
*CU Denver*	16	5
*CU Boulder*	20	6.2
*CU Anschutz*	254	78.9
*Colorado State University*	16	5
*Rocky Mountain Regional VA Medical Center*	7	2.2
**What stage are these projects on the clinical and translational research spectrum?** (*n* = 306)*		
*Translation to Animal Models (T0.5)*	21	6.9
*Translation to Humans (T1)*	42	13.7
*Translation to Patients (T2)*	48	15.7
*Translation to Practice (T3)*	74	24.2
*Translation to Population (T4)*	36	11.8
*Don’t know*	85	27.8
**Prior to the remote work recommendation for COVID-19, how many days a week did you work from home?** (*n* = 322)		
*0 days*	163	50.6
*1 day*	83	25.8
*2 days*	36	11.2
*3 days*	10	3.1
*4 days*	6	1.9
*5 days*	17	5.3
*6–7 days (Monday–Friday, plus some weekends)*	7	2.2
**To what extent does remote work during COVID-19 interfere with your ability to conduct your research activities?** (*n* = 322)		
*Does not interfere*	78	24.2
*Interferes somewhat*	144	44.7
*Interferes to a great extent*	100	31.1
**Will you be stopping any research during the COVID-19 pandemic?** (*n* = 322)		
*None*	62	19.3
*Some*	178	55.3
*All*	53	16.5
*Not applicable*	29	9
**Who else is in your home during the day and/or night (check all that apply)?** (*n* = 322)		
*Live alone*	21	6.5
*Spouse/partner*	260	80.7
*Roommate*	21	6.5
*Children+*	151	46.9
*Parents*	20	6.2
*Pets*	131	40.7
*Other people in house*	15	4.7
**Ages of children in the home (** *n* = 217) *		
*<12 months*	9	2.8
*Toddlers (13 months–3 years)*	28	8.7
*Pre-school (3–4 years)*	22	6.8
*Elementary (5–10 years)*	59	18.3
*Middle School (11–13 years)*	38	11.9
*High School (14–18 years)*	37	11.5
*College Age (19–21 years)*	18	5.5
*Adult Children (22+)*	6	1.9

CCTSI, Colorado Clinical and Translational Science Institute.

+ Number of respondents reporting children in the home * Sample includes families with more than one child in the home. *T.05: *Basic research; T1: Preclinical studies (phase 1 trials); T2: Clinical efficacy and effectiveness (Phase 2–3 trials); T3: Translation to practice, health services, dissemination and implementation research; T4; population level outcomes research for global impact.*

Very few respondents reported a history of remote work experience, with 51% (*n* = 163) indicating they had never worked remotely and 26% (*n* = 83) reported working remotely only 1 day a week prior to the pandemic. Only 24% (*n* = 76) of respondents indicated they had previously worked from home >2 days a week (Table 1). There was no significant association between remote workdays prior to COVID-19 and educational degree, professional degree, gender, research role, faculty investigator position, or transitional research spectrum. An association was noted between prior remote workdays and reported interference with research (*P* = 0.018) and stopping of research (*P* = 0.026). (Supplemental Material 1)

### Interference of Remote Work on Research Activities

Remote work during COVID-19 was reported by 45% (*n* = 144) of participants to somewhat interfere with their program of research. For 31% (*n* = 100), remote work interfered to a great extent. Interference with research activities was reported mostly by those with an educational degree of a PhD (*P* = 0.005), a professional degree of a medical doctor (*P* = 0.025), a research role of a faculty investigator (*P* = 0.037), and transitional research spectrum of Translation to Patients (*P* < 0.001). A relationship was noted between those who reported interference with research activities and plans to stop some research (*P* < 0.001) (Table 2).

**Table 2.** All survey responses stratified by level of reported interference with research activities

Questions & Responses	Does Not Interfere	Interferes Somewhat	Interferes to a Great Extent	
	(*n* = 78)	(*n* = 144)	(*n* = 100)	
	*n* (%)	*n* (%)	*n* (%)	*P*-value
**Educational Degree**	*n* = 78	*n* = 144	*n* = 99	**0.005**
*Associates*	0 (0)	1 (0.7)	0 (0)	
*Bachelors*	12 (15.4)	16 (11.3)	18 (18.2)	
*Masters*	29 (37.2)	39 (27.7)	11 (11.1)	
*PhD*	27 (34.6)	60 (42.6)	52 (52.5)	
*Practice Doctorate (MD, PharmD, JD)*	10 (12.8)	25 (17.7)	18 (18.2)	
**Professional Degree**	*n* = 22	*n* = 50	*n* = 29	**0.025**
*Registered Nurse or Advanced Practice Nurse*	1 (4.5)	3 (6.0)	6 (20.7)	
*Medical Doctor/Doctor of Osteopathy*	11 (50.0)	28 (56.0)	20 (68.9)	
*Dietician/Social Worker/Pharmacist*	2 (9)	3 (6.0)	2 (6.8)	
*Public Health*	8 (36.4)	16 (32.0)	1 (3.4)	
**Gender**	*n* = 78	*n* = 144	*n* = 100	0.374
*Male*	20 (25.6)	30 (20.8)	29 (29.0)	
*Female*	58 (74.4)	112 (77.8)	70 (70.0)	
*Non-binary*	0 (0)	0 (0)	1(1.0)	
*Prefer not to answer*	0 (0)	2 (1.4)	0 (0)	
**Research Role**	*n* = 77	*n* = 144	*n* = 100	**<0.001**
*Faculty investigator*	26 (33.8)	76 (52.8)	63 (63.0)	
*Non-faculty investigator*	0 (0)	13 (9.0)	11 (11.0)	
*Research support staff*	1 (1.3)	3 (2.1)	1 (1.0)	
*Research administration*	16 (20.8)	5 (3.5)	1 (1.0)	
*Research clinical staff*	34 (44.2)	47 (32.6)	24 (24.0)	
**Faculty Investigator**	*n* = 26	*n* = 76	*n* = 62	**0.037**
*Instructor/Senior Instructor*	2 (7.7)	11 (14.5)	1 (1.6)	
*Assistant Professor*	8 (30.8)	27 (35.5)	32 (51.6)	
*Associate Professor*	5 (19.2)	22 (28.9)	15 (24.2)	
*Professor*	10 (38.5)	15 (19.7)	13 (21.0)	
*Other*	1 (3.8)	1 (1.3)	1 (1.6)	
Translational Research Spectrum*	*n* = 72	*n* = 137	*n* = 97	**<0.001**
*Translation to Animal Models (T0.5)*	0 (0)	7 (5.1)	14 (14.4)	
*Translation to Humans (T1)*	8 (11.1)	14 (10.2)	20 (20.6)	
*Translation to Patients (T2)*	6 (8.3)	21 (15.3)	21 (21.6)	
*Translation to Practice (T3)*	17 (23.6)	43 (31.4)	14 (14.4)	
*Translation to Population (T4)*	11 (15.3)	18 (13.1)	7 (7.2)	
*Don’t know*	30 (41.7)	34 (24.8)	21 (21.6)	
**Previous Remote Workdays**	*n* = 78	*n* = 144	*n* = 100	0.2304
*0 days*	36 (46.2)	66 (45.8)	61 (61)	
*1 day*	16 (20.5)	43 (29.9)	24 (24.0)	
*2 days*	14 (17.9)	16 (11.1)	6 (6.0)	
*3 days*	4 (5.1)	4 (2.8)	10 (3.1)	
*4 days*	2 (2.6)	2 (1.4)	2 (2.0)	
*5 days*	5 (6.4)	9 (6.3)	3 (3.0)	
*6–7 days (Monday–Friday, some weekends)*	1 (1.3)	4 (2.8)	2 (2.0)	
**Stopping of Research**	*n* = 78	*n* = 144	*n* = 100	**<0.001**
*None*	36 (46.2)	23 (16)	3 (3.0)	
*Some*	26 (33.3)	95 (66.0)	57 (57.0)	
*All*	0 (0)	15 (10.4)	38 (38.0)	
*Not applicable*	16 (20.5)	11 (7.6)	2 (2.0)	

Associations calculated using Fisher’s Exact Test. Statistical significance set at *P* < 0.05.

When the sample was limited to only female respondents (*n* = 240), remote work during COVID-19 was reported by 47% (*n* = 112) of participants to somewhat interfere with their program of research. For 29% (*n* = 70), remote work interfered to a great extent. Interference with research activities continued to be reported by those with a PhD (*P* = 0.031), medical doctors (*P* = 0.037), a research role of a faculty investigators (*P* < 0.001), and transitional research spectrum of Translation to Patients (*P* < 0.001). The relationship between those who reported interference with research activities and plans to stop some research persisted in this population (*P* < 0.001) (Table 3).

**Table 3.** Female survey responses stratified by level of reported interference with research activities

Questions & Responses	Does Not Interfere	Interferes Somewhat	Interferes to a Great Extent	
	(*n* = 58)	(*n* = 112)	(*n* = 70)	
	*n* (%)	*n* (%)	*n* (%)	*P*-value
**Educational Degree**	*n* = 58	*n* = 111	*n* = 69	**0.031**
*Associates*	0 (0)	0 (0)	0 (0)	
*Bachelors*	10 (17.2)	15 (13.5)	14 (20.3)	
*Masters*	22 (37.9)	32 (28.8)	9 (13)	
*PhD*	20 (34.5)	43 (38.7)	36 (52.2)	
*Practice Doctorate (MD, PharmD, JD)*	6 (10.3)	21 (18.9)	10 (14.5)	
**Professional Degree**	*n* = 15	*n* = 43	*n* = 18	**0.037**
*Registered Nurse or Advanced Practice Nurse*	1 (6.7)	3 (7)	6 (33.3)	
*Medical Doctor/Doctor of Osteopathy*	6 (40)	23 (53.4)	10 (55.6)	
*Dietician/Social Worker/Pharmacist*	2 (13.3)	3 (7)	1 (5.6)	
*Public Health*	6 (40)	14 (32.6)	1 (5.6)	
**Research Role**	*n* = 57	*n* = 112	*n* = 70	**<0.001**
*Faculty investigator*	17 (29.8)	56 (50)	39 (55.7)	
*Non-faculty investigator*	0 (0)	10 (8.9)	10 (14.3)	
*Research support staff*	1 (1.8)	1 (0.9)	1 (1.4)	
*Research administration*	13 (22.8)	5 (4.5)	1 (1.4)	
*Research clinical staff*	26 (45.6)	40 (35.7)	19 (27.1)	
**Faculty Investigator**	*n* = 17	*n* = 56	*n* = 38	0.226
*Instructor/Senior Instructor*	1 (5.9)	9 (16.1)	1 (2.6)	
*Assistant Professor*	7 (41.2)	20 (35.7)	21 (55.3)	
*Associate Professor*	4 (23.5)	15 (26.8)	9 (22.7)	
*Professor*	4 (23.5)	12 (21.4)	6 (15.8)	
*Other*	1 (5.9)	0 (0)	1 (2.6)	
**Translational Research Spectrum**	*n* = 53	*n* = 107	*n* = 68	**<0.001**
*Translation to Animal Models (T0.5)*	0 (0)	5 (5.8)	7 (10.3)	
*Translation to Humans (T1)*	4 (7.5)	13 (12.1)	15 (22.1)	
*Translation to Patients (T2)*	4 (7.5)	16 (15)	15 (22.1)	
*Translation to Practice (T3)*	14 (26.4)	34 (31.8)	11 (15.9)	
*Translation to Population (T4)*	8 (15.1)	15 (14)	6 (8.8)	
*Don’t know*	23 (43.4)	24 (22.4)	14 (20.6)	
**Previous Remote Workdays**	*n* = 58	*n* = 112	*n* = 70	0.054
*0 days*	24 (41.4)	50 (44.6)	47 (67.1)	
*1 day*	14 (24.1)	38 (33.9)	12 (17.1)	
*2 days*	11 (19)	11(9.8)	5 (7.1)	
*3 days*	3 (5.2)	3 (2.7)	1 (1.4)	
*4 days*	1 (1.7)	2 (1.8)	2 (2.9)	
*5 days*	5 (8.6)	7 (6.3)	2 (2.9)	
*6–7 days (Monday–Friday, some weekends)*	0 (0)	1 (0.9)	1 (1.4)	
**Stopping of Research**	*n* = 58	*n* = 112	*n* = 70	**<0.001**
*None*	22 (37.9)	16 (14.3)	2 (2.9)	
*Some*	22 (37.9)	77 (68.8)	40 (57.1)	
*All*	0 (0)	12 (10.7)	27 (38.6)	
*Not applicable*	14 (24.1)	7 (6.3)	1 (1.4)	

### Plans to Slow or Stop Research Activities

Over half of participants (*n* = 178; 55%) reported they would be stopping some of their research due to COVID-19 and/or remote work, while 16% (*n* = 53) reported stopping all research activities. Participants were asked to describe why their research was being put on hold. Of the 224 open text responses, the most frequently noted reasons for stopping research included not being able to conduct face-to-face visits and testing with study participants due to safety concerns, not being able to work remotely with animals, cell lines, and biologicals in laboratories, and that community partners were focused on the COVID-19 response and were not available to participate in research. Additional reasons included financial issues and staff being redistributed to work on studies that related to the COVID-19 response, restriction on hiring and use of student workers, and the travel ban placed by Universities and other employers.

### Barriers to Remote Work

The primary barrier to remote work during COVID-19 was participants missing daily face-to-face interaction (work and social) with colleagues (*n* = 198; 62%). Additional barriers included the absence of daily routines (*n* = 137; 43%), children in the home (*n* = 117; 36%), limited private workspace in the home (*n* = 108; 34%), and internet issues (*n* = 81; 25%). These barriers were reported to be a daily issue for 44% (*n* = 141) of participants (Table 4). Analysis conducted on 74 free text responses in the “other” field for the question “What barriers to remote work are you experiencing?” revealed additional barriers. These included: (1) personnel management barriers related to hiring, working, supervising, efficient, and/or effective collaboration; (2) workspace barriers related to the home office setting versus campus office; (3) research specific barriers including lack of access to subjects, data, materials, and collaborators; (4) personal and professional impacts of the pandemic (e.g. stress, loneliness, isolation, absence of support from leadership, new fiscal challenges, loss of productivity); and (5) barriers to clinical practice and teaching. The “other” barrier codes, definitions, and counts are organized by level of interference with research categories and presented with representative text responses in Supplemental Material 2.

**Table 4.** Barriers to remote work and strategies to engage staff

**What barriers to remote work are you experiencing? Check all that apply** (*n* = 322)	*N*	%
*Missing daily face-to-face interaction (work/social) with colleagues*	198	61.5
*Absence of daily routine*	137	42.5
*Children in home*	117	36.3
*Limited private workspace in home*	108	33.5
*Internet issues*	81	25.2
*Other (See Supplemental Material 2)*	75	23.3
*Inadequate IT equipment in home*	48	14.9
*No barriers*	46	14.3
*Secure VPN connection issues*	39	12.1
*Not permitted to remote work*	19	5.9
*Elder care*	10	3.1
**How frequently do these barriers impact your work?** (*n* = 322)		
*Rarely*	36	11.2
*Once a week*	38	11.8
*2–3 times a week*	74	23
*Daily*	141	43.8
*Not applicable*	33	10.2
**What strategies are being implemented by local leadership, investigators, project leads, or project managers to engage staff in a productive way (check all that apply)?** (*n* = 322)		
*Videoconference meetings*	283	87.9
*Altered timelines and project expectations*	180	55.9
*Informal videoconference-based gatherings (coffee, lunch, social)*	142	44.1
*Daily COVID email updates*	81	25.2
*Group self-care activities (on-line meditation, knitting, book club)*	63	19.6
*Daily email updates*	61	18.9
*Daily huddles via phone or video chats*	45	14
*Group text updates*	31	9.6
*None of these are implemented in my team(s)*	16	5

### Engagement Strategies to Support Staff Productivity

The majority of participants indicated they had started videoconference meetings (*n* = 283; 88%) and had altered timelines and project expectations for themselves and their teams (*n* = 180; 56%). Many started informal videoconference-based gatherings such as coffee breaks, lunch, and end-of-week social times (*n* = 142; 44%) or group self-care activities such as online meditation, knitting, or book clubs (*n* = 63; 20%). Communication strategies varied from daily COVID-19 email updates (*n* = 81; 25%), daily team or research email updates (*n* = 61; 19%), daily huddles via phone or video chat (*n* = 45; 14%) to group text updates (*n* = 31; 10%). Only 5% (*n* = 16) of participants reported that no new strategies had been implemented during COVID-19 (Table 2).

### Workarounds to Remote Work Barriers

Analyses of the 245 open text responses identified seven common workarounds: (1) The use of videoconferencing technology for meetings, data collection, and socialization; (2) increased communication and frequency of meetings; (3) prioritizing work that can be done remotely; (4) developing coping strategies to working remotely from home; (5) using available information technology remote access tools; (6) creating a home office space and routine; (7) and working around childcare responsibilities in the home. Some respondents indicated there were no workarounds available to address their barriers. The workaround codes, definitions, and counts are organized by level of interference with research categories and presented with representative text responses in Supplemental Material 3.

## Discussion

We conducted a survey of CCTSI scientists and staff to systematically collect, analyze, and share remote work experiences, barriers, and strategic workarounds created by CCTSI members during the first months of the COVID-19 pandemic response in Colorado. The results indicate that within the first 6 weeks of the stay-at-home order, many were experiencing significant interference with their research activities. This resulted in the slowing or stopping of programs of research. One of the primary barriers identified was that few survey respondents had worked remotely prior to COVID-19, suggesting they did not have the home office equipment, separate workspace, rituals and routines [18], high-speed internet, or familiarity with remote IT and conferencing tools that are required to support remote work [19]. Respondents shared that once they built their home office infrastructure, developed rituals to manage their day, and received guidance and support from department, University or hospital leadership, the challenge became connecting and collaborating with colleagues.

Over 60% of respondents reported they missed the daily face-to-face work and social interaction with colleagues. The chance meetings and serendipitous interactions, the coffee breaks, the mentoring and brainstorming that occur in the office support socialization and sensemaking. Further, these interactions are sources of new ideas for many scientists. Research teams reported moving their meetings quickly to interactive technology platforms supported by their organizations, including Zoom and Microsoft Teams to support engagement and social interaction. Daily huddles and discussion of timelines and expectations increased accountability within teams while providing individuals with the autonomy and flexibility to adapt their work. Moving forward, CCTSI, University, and hospital leaders should promote system-wide use of these technology platforms and teamwork strategies and provide training and tech support to ensure all scientists and staff are set up for success.

Remote work makes social interaction challenging, which is why some in our sample are reporting feelings of isolation and loneliness. This is concerning given lonely workers can experience lower performance [20], quit more often [21], and feel less satisfied with their jobs [22]. Though many respondents reported they “*truly enjoy working from home.*” Others wrote they were having to “*work 50-hour weeks AND doing 40–50 hours of childcare/schooling each week*” and were experiencing a “*never ending workday… the boundaries between work and home life become fuzzy*.” Many reported they “*feel exhausted by the daily routine*,” “*feel more tired now and [are] having a harder time focusing*” with one person reporting they have “*increased their anxiety medicine*.” Many were “*not optimistic*” they could keep up their focus and level of productivity if remote work continued past the summer. This was especially prevalent for women who reported childcare responsibilities.

In our sample, associations were noted for female respondents and the level of interference and plans to stop research. The scientific literature has documented the unequal effects of the COVID-19 pandemic on female scientists in general, and those with young children in particular [10,23,24]. In one study, the proportion of women publishing papers in medical journals as first author dedicated to the COVID-19 pandemic was 19% lower than for papers published in the same journals in 2019 [25]. In a second study of non-medical principal investigators in the US and Europe, being a woman with young children was the biggest predictor of research disparities during COVID-19 [23]. Female respondents in our survey indicated they “*have no childcare available to us and we are in charge of homeschooling our children,*” plus “*on top of all this, we have increased household duties from being home all the time (dishes, cleaning, etc.).”* and “*I am in mom-mode 24/7 now.*” Though not assessed in this study, the career cost of COVID-19 to female researchers may be high [9,24]. Although fathers are not immune to the impact of remote work during COVID-19, it is traditionally women who are responsible for unpaid care and domestic work in home and communities [24].

Scientific fields have been affected differently by remote work during COVID-19 [23,26]. In our sample, those who rely on physical laboratories, time-sensitive experiments, and interaction with patients or the community reported the largest impact. Unfortunately, many respondents did not know where their research fell on the clinical and translational spectrum. This may be due to the absence of explicit definitions in the survey. How the COVID-19 pandemic will impact certain types of research and scientific outputs, which include publications and new grant submissions is of great concern. Recent reports have noted a decrease in the proportion of scientific publication submissions by female and early career scientists from March and April of 2019 to the same months in 2020 [9]. The National Institutes of Health reported that for the June 2020 grant cycle, the number of applications was 10% higher than the same time last year [26]. The proportion of applications in which the principal investigator was a woman remained stable [26]. These data offer a first look into the effects of the pandemic. However, these publications and research projects were most likely in progress prior to COVID-19 remote work. The many months of lab slowdowns, enforced remote work and ongoing school, daycare, and camp closures may result in long-term detrimental effects on researchers, staff, and the scientific endeavor [26].

To mitigate the negative impacts of remote work during COVID-19, survey respondents shared physical, technological, and relational strategies and workarounds to enhance collaboration and resiliency. These included the use of videoconferencing and team collaboration platforms which can support the communication, psychosocial, and informational needs of individuals and teams [7,27,28]. Though “Zoom fatigue” is real [29], methods to bring some sense of control to the remote work experience exist. Some teams initiated daily huddles to maintain focus, purpose, and casual interaction between staffs. Others used meeting time to build social-emotional health by sharing gratitude, role modeling generosity, and encouraging self-care activities. Other strategies include limiting meeting times, minimizing screen time by writing instead of typing, not always opting for video calls, taking tech-free breaks, and moving around as much as possible. These strategies can be implemented by individuals but sustainability is enhanced when these strategies are supported by department and organizational leadership.

Limitations of our study were the relatively small respondent sample, predominance of female respondents, and self-report nature of the survey. We did not ask respondents how much research funding they currently receive, the source of funding, the percent of their time dedicated to research or their research setting. Further, we could not study the impact of remote work on each stage of the clinical and translational spectrum. Due to this, our findings may not reflect the views or beliefs of all CCTSI scientists and staffs, it may have included those with low research activity, and did not enhance our understanding of how remote work is impacting research on different levels of the clinical and translational spectrum. Because of these limitations, the study findings may not be generalizable to other clinical and translational scientists and staff. However, the results, lessons learned, and study limitations can inform other institutions as they develop surveys and assess plans to address needs of clinical and translational scientists and staff in conducting remote work. Study findings must be interpreted within this context.

## Conclusions

This study identified multiple strategies and workarounds being used to support engagement and productivity during COVID-19 remote work. Overall, CCTSI scientists shared they are approaching the dynamic challenges of working, parenting, and coping during a worldwide pandemic with agility and self-compassion. Respondents felt this approach would help them maintain their mental and physical health so as to persevere during a time of great uncertainty. As scientists and research organizations settle into remote work, these insights can help design improved systems to support communication, relationships, productivity, and collective coping during the COVID-19 pandemic.

## Supplementary material

For supplementary material accompanying this paper visit http://dx.doi.org/10.1017/cts.2020.570. click here to view supplementary material

## Data Access, Responsibility, Analysis

Dr. Gilmartin, Ms. Connelly and Ms. Hebbe had full access to all the data in the study and take responsibility for the integrity of the data and the accuracy of the data analysis. Dr. Gilmartin of the Denver/Seattle Center of Innovation for Veteran-Centered and Value Driven Care conducted and is responsible for the data analysis.

## Data Sharing Statement

The data is available to researchers with CCTSI membership.

## Statistical Code

Available to interested readers by contacting Dr. Gilmartin.

## Protocol

Available to interested readers at Mountain Scholar https://mountainscholar.org/handle/10968/5619

## References

[1] Ghebreyesus TA . WHO director-General’s opening remarks at the media briefing on COVID-19. World Health Organization [Internet], 2020 [cited March 11, 2020]. (https://www.who.int/dg/speeches/detail/who-director-general-s-opening-remarks-at-the-media-briefing-on-covid-19---11-march-2020)

[2] North American companies take steps to protect employees from coronavirus epidemic. WillisTowersWatson [Internet], 2020 [cited March 5, 2020]. (https://www.willistowerswatson.com/en-US/News/2020/03/north-american-companies-take-steps-to-protect-employees-from-coronavirus-epidemic)

[3] Polis J . Gov. Polis Announces Statewide Stay-At-Home Order, Provides Update on Colorado Response to COVID-19. State of Colorado [Internet], 2020 [cited March 25, 2020]. (https://www.colorado.gov/governor/news/gov-polis-announces-statewide-stay-home-order-provides-update-colorado-response-covid-19)

[4] Johnson MO , Suskewicz J . Does your company have a long-term plan for remote work? Harvard Business Review Web site [Internet], 2020 [cited July 20, 2020]. (https://hbr.org/2020/07/does-your-company-have-a-long-term-plan-for-remote-work)

[5] Primack RB , Setash C . COVID-19 is eroding scientific field work - and our knowledge of how the world is changing. The Conversation Web site [Internet], 2020 [cited May 19, 2020]. (https://theconversation.com/covid-19-is-eroding-scientific-field-work-and-our-knowledge-of-how-the-world-is-changing-137045)

[6] Powell K . Science-Ing from home. Nature 2020; 5807(7803): 419–421. 10.1038/d41586-020-00935-3 32218550

[7] Larson BZ , Makarius EE , Vroman SR. A guide to managing your (Newly) remote workers. Harvard Business Review 2020; 18:1–6.

[8] Impey C . Coronavirus: Social Distancing is Delaying Vital Scientific Research. The Conversation [Internet], 2020 [cited March 18, 2020]. (https://theconversation.com/coronavirus-social-distancing-is-delaying-vital-scientific-research-133689)

[9] Viglione G . Are women publishing less during the pandemic? Here’s what the data say. Nature 2020; 581(7809): 365–366. 32433639 10.1038/d41586-020-01294-9

[10] Minello A . The pandemic and the female academic. Nature 2020; 17: 2020. 10.1038/d41586-020-01135-9 32303729

[11] Harris PA , Taylor R , Minor BL , et al. The REDCap consortium: building an international community of software platform partners. Journal of Biomedical Informatics 2019; 95: 103208. 31078660 10.1016/j.jbi.2019.103208 PMC7254481

[12] Gilmartin H . Survey CCTSI Remote Work During COVID-19. Mountain Scholar [Internet], 2020 [cited September 4, 2020]. (https://mountainscholar.org/handle/10968/5619)

[13] Staples DS . A study of remote workers and their differences from non-remote workers. Journal of Organizational and End User Computing (JOEUC) 2001; 13(2): 3–14.

[14] Felstead A , Henseke G . Assessing the growth of remote working and its consequences for effort, well-being and work-life balance. New Technology, Work and Employment 2017; 32(3): 195–212.

[15] Kahana J . 9 Principles to Better Remote Work. Medium [Internet], 2020 [cited March 13, 2020]. (https://www.medium.com/caveday/9-principles-to-working-better-remotely-a61aace1d513)

[16] Translational Science Spectrum. National Institutes of Health [Internet], 2020 [cited August 16, 2020]. (https://www.ncats.nih.gov/translation/spectrum)

[17] Bengtsson M . How to plan and perform a qualitative study using content analysis. NursingPlus Open 2016; 2: 8–14.

[18] Kahana J . 7 Ways to Recreate a Cave in Your Office. Medium [Internet], 2018 [cited June 27, 2020]. (https://www.medium.com/caveday/7-ways-to-recreate-a-cave-in-your-office-b1583536dbe1)

[19] Neeley T. 15 questions about remote work, answered. Harvard Business Review 2020: 16. https://hbr.org/2020/03/15-questions-about-remote-work-answered. Accessed June 27, 2020

[20] Ozcelik H , Barsade S . Work loneliness and employee performance. Paper presented at: Academy of management proceedings, 2011.

[21] Ertosun ÖG , Erdil O . The effects of loneliness on employees’ commitment and intention to leave. Procedia-Social and Behavioral Sciences 2012; 41: 469–476.

[22] Berinato S. What Do We Know About Loneliness and Work? Harvard Business Review. Connecting at Work Web site [Internet], 2017 [cited Aug 10, 2020]. (https://www.hbr.org/2017/09/what-do-we-know-about-loneliness-and-work)

[23] Myers KR , Tham WY , Yin Y , et al. Unequal effects of the COVID-19 pandemic on scientists. Nature Human Behaviour 2020; 4(9): 880–883. 10.1038/s41562-020-0921-y 32669671

[24] Gewin V . The career cost of COVID-19 to female researchers, and how science should respond. Nature 2020; 583: 867–869. 32690964 10.1038/d41586-020-02183-x

[25] Andersen JP , Nielsen MW , Simone NL , Lewiss RE , Jagsi R . Meta-research: COVID-19 medical papers have fewer women first authors than expected. Elife 2020; 9(e58807): 1–7. 10.7554/eLife.58807 PMC7304994 32538780

[26] Lauer M . An Early Look at Applications Submitted During the Pandemic. National Insitutes of Health. Open Mike: Extramural Nexus Web site [Internet], 2020 [cited July 28, 2020]. (https://www.nexus.od.nih.gov/all/2020/07/28/an-early-look-at-applications-submitted-during-the-pandemic/)

[27] Vogus TJ , Iacobucci D . Creating highly reliable health care: how reliability-enhancing work practices affect patient safety in hospitals. ILR Review 2016; 69(4): 911–938.

[28] Gittell JH . Relationshps and resilience: care provider responses to pressures from managed care. The Journal of Applied Behavioral Science 2008; 44(1): 25–47.

[29] Fosslien L , Duffy MW . How to combat zoom fatigue. Harvard Business Review Web site [Internet], 2020 [cited April 29, 2020]. (https://www.hbr.org/2020/04/how-to-combat-zoom-fatigue)

