# Supplementary material for: Assessing the impact of remote work during COVID-19 on clinical and translational scientists and staff in Colorado
Source: J Clin Transl Sci. 2020 Dec 21;5(1):e71. doi: 10.1017/cts.2020.570 (PMC8027555; doi:10.1017/cts.2020.570)
Supplement: Supplementary file 1 [file S2059866120005701sup001.docx]

**Supplemental Material 1**

**Association Between Previous Remote Workdays and Study Demographics**

|  | 0-1 Previous Remote Workdays  (N=246) | 2-7 Previous Remote Workdays  (N=76) |  |
| --- | --- | --- | --- |
| **Questions** & *Responses* |  | |  |
|  | N (%) | N (%) | p.value |
| **Interference**  *Does not Interfere*  *Interferes Somewhat*  *Interferes to a Great Extent* | N=246  52 (21.1)  109 (44.3)  85 (34.6) | N=76  26 (34.2)  35 (46.1)  15 (19.7) | **0.018** |
| **Educational Degree**  *Associates*  *Bachelors*  *Masters*  *PhD*  *Practice Doctorate (MD, PharmD, JD)* | N=244  1 (0.4)  40 (6.1)  61 (25)  99 (40.6)  43 (17.6) | N=74  0 (0)  6 (8.1)  18 (24.3)  40 (54.1)  10 (13.5) | 0.191 |
| **Professional Degree**  *Registered Nurse or Advanced Practice Nurse*  *Medical Doctor/Doctor of Osteopathy*  *Dietician/Social Worker/Pharmacist*  *Public Health* | N=84  8 (9.5)  49 (58.3)  6 (7.1)  21 (25) | N=16  2 (12.5)  10 (62.5)  1 (6.3)  3 (18.8) | 0.843 |
| **Gender**  *Male*  *Female*  *Non-binary*  *Prefer not to answer* | N=246  59 (24)  185 (75.2)  1 (0.4)  1 (0.4) | N=76  20 (26.3)  55 (72.4)  0 (0)  1 (1.3) | 0.586 |
| **Research Role**  *Faculty investigator*  *Non-faculty investigator*  *Research support staff*  *Research administration*  *Research clinical staff* | N=245  125 (51)  16 (6.5)  2 (0.8)  20 (8.2)  82 (33.5) | N=76  40 (52.6)  8 (10.5)  3 (3.9)  2 (2.6)  23 (30.3) | 0.092 |
| **Faculty Investigator**  *Instructor/Senior Instructor*  *Assistant Professor*  *Associate Professor*  *Professor*  *Other* | N= 124  11 (8.9)  56 (45.2)  30 (24.2)  26 (21)  1 (0.8) | N=40  3 (7.5)  11 (27.5)  12 (30)  12 (30)  2 (5) | 0.115 |
| **Translational Research Spectrum ***  *Translation to Animal Models (T0.5)*  *Translation to Humans (T1)*  *Translation to Patients (T2)*  *Translation to Practice (T3)*  *Translation to Population (T4)*  *Don’t know* | N=235  17 (7.2)  33 (14)  39 (16.6)  51 (21.7)  29 (12.3)  66 (28.1) | N=71  4 (5.6)  9 (12.7)  9 (12.7)  23 (32.4)  7 (9.9)  19 (26.8) | 0.649 |
| **Stopping of Research**  *None*  *Some*  *All*  *Not applicable* | N=246  43 (17.5)  135 (54.9)  48 (19.5)  20 (8.1) | N=76  19 (25)  43 (56.6)  5 (6.6)  9 (11.8) | **0.026** |

**Key: ***T.05: *Basic research; T1: Preclinical studies (phase 1 trials); T2: Clinical efficacy and effectiveness (Phase 2-3 trials); T3: Translation to practice, health services, dissemination and implementation research; T4; population level outcomes research for global impact*

**Supplemental Material 2**

**Other Barriers to Remote Work: Coded Categories**

| Codes | Definition | Interference with Research Categories | Count | Example of Text Responses |
| --- | --- | --- | --- | --- |
| Barrier to working remotely | Barrier related to hiring, supervising, effective and/or efficient collaboration | None | 1 | *On-the-job training[is] facilitated by face-to-face communication* |
|  |  | Somewhat | 10 | *…less efficient communication due to lack of in-person interacting with colleagues*  *...more video meetings, more daily check-ins. It's more contact than I've ever had with my teams.* |
|  |  | Great Extent | 8 | *Trying to figure out things for PRAs and others to do without being able to do new experiments.* |
| Barrier to home office setting | Barrier related to home office setting versus campus office | None | 5 | *Physical discomfort … sitting down so much more…* |
|  |  | Somewhat | 12 | *Lack of access to work computer-working from personal laptop difficult…*  *… Lack of childcare/schooling resulting in interrupted and disjointed work hours* |
|  |  | Great Extent | 4 | *No adequate work furniture like a desk* |
| Barrier to conducting research | Barrier to conduct of research activities | None | 0 |  |
|  |  | Somewhat | 20 | *…had to discontinue a clinical trial because it involved seeing patients in person*  *…lack of access to the lab* |
|  |  | Great Extent | 21 | *...can't gather any new data*  *…need to do work in hospital... research in the division has been suspended* |
| Impact of pandemic (personal) | Personal issues specific to COVID-19 pandemic | None | 3 | *Never ending workday.*  *...boundaries between work and home life become fuzzy* |
|  |  | Somewhat | 2 | *I live alone and was fairly isolated before the pandemic, so not being able to go to work and interact with coworkers and study subjects as normal has been very difficult. My mental health is definitely suffering*  *…being in an uncomfortable working space with no books or routine or colleagues to engage with I've felt isolated and my work has suffered a bit.* |
|  |  | Great Extent | 6 | *Constantly changing guidance is stressful for the whole team*  *… everyone is frustrated and afraid of layoffs or other possible changes to employment status* |
| Impact of pandemic (professional) | Professional issues specific to COVID-19 pandemic | None | 0 |  |
|  |  | Somewhat | 7 | *…may fall farther behind than non-clinicians right now…* |
|  |  | Great Extent | 3 | *…will lose some patients...may lose interest from having to be put [research] on "hold"* |
| Barriers to clinical practice | Barrier to clinical care by academic research clinician | None | 0 |  |
|  |  | Somewhat | 1 | *…changes to clinical shifts- increased hours, many and ongoing changes to hours/locations…* |
|  |  | Great Extent | 3 | *… although my clinical responsibilities have decreased, the time it takes to conduct my clinical responsibilities has increased…* |
| Barriers to teaching | Barrier to remote teaching of students | None | 0 |  |
|  |  | Somewhat | 0 |  |
|  |  | Great Extent | 2 | *When campus was closed, and all teaching moved to remote learning there was zero support for our students who did not have the capability to learn remotely.* |

**Supplemental Material 3**

Workarounds Created to Address Barriers to Remote Work

| Codes | Definition | Interference with Research Categories | Count | *Example of Text Responses* |
| --- | --- | --- | --- | --- |
| Videoconference meetings | Workaround is use of video conferencing technology | None | 28 | *Zoom colleague meet ups, Microsoft Meetings...Zoom Office Hours* |
|  |  | Somewhat | 53 | *Zoom meetings have been the most important way to stay connected and engaged with research teams.* |
|  |  | Great Extent | 22 | *Zooming constantly* |
| Increased communication/ frequency of meetings | Workaround is increasing communication and frequency of meetings | None | 18 | *Daily check-in with staff…* |
|  |  | Somewhat | 44 | *Daily "huddles", messaging through [Microsoft] Teams*  *Daily work summaries emailed to director for increased transparency and communication.* |
|  |  | Great Extent | 26 | *Weekly scheduling meeting to outline tasks/goals for the week* |
| Conduct work that can be done remotely | Workaround is conducting work that can be done remotely | None | 1 | *I have delineated expectations for publication, grant submission (from trainees)* |
|  |  | Somewhat | 24 | *Focus on regulatory work, which does not require patient interaction or access to sensitive records* |
|  |  | Great Extent | 22 | *Writing papers and grants while clinical trials are on hold*  *Have shifted emphasis to analysis of pre-existing data rather than acquisition of new data.* |
| Coping Strategies | Workaround is developing coping strategies to working remotely/from home | None | 4 | *… more lenient response times* |
|  |  | Somewhat | 22 | *… ask for help when needed*  *limiting non-essential activities.* |
|  |  | Great Extent | 9 | *Aiming for patience and grace.* |
| Use IT remote access tools | Workaround is to use available IT remote access tools | None | 2 | *Log in at alternate times - early morning or evening*  *I work outside my hours … when VPN connection isn’t slow.* |
|  |  | Somewhat | 15 | *Been given additional tech support and international access*  *Learning how to digitally sign things so they don't need to be printed* |
|  |  | Great Extent | 4 | *Able to access files via VPN* |
| Create home office space/routine | Workaround is to create a home office space and routine | None | 17 | *Bought a new monitor, borrowed a back pillow and headphones*  *My mentor bought me another monitor, keyboard, and mouse*  *I upgraded my internet access at home.* |
|  |  | Somewhat | 37 | *… set up somewhat private workspace in bedroom*  *…get up very early, work weekends* |
|  |  | Great Extent | 13 | *Our office allowed us to bring our desktop computers, monitors, and accessories home.*  *Created a separate space for an office at home….* |
| Children focused workaround | Workarounds due to childcare responsibilities in home | None | 1 | *My husband and I take turns with the kids* |
|  |  | Somewhat | 17 | *…Moving meetings to times when young children doing remote learning are able to do independent work* |
|  |  | Great Extent | 4 | *For me, survival mode with my spouse. Our young children are the priority, and work happens with "shifts" and late at night.* |
| Cannot work around barriers | No workaround to existing barriers | None | 0 |  |
|  |  | Somewhat | 1 | *…the sheer volume of people working from home is impacting the VPN strength* |
|  |  | Great Extent | 9 | *We have not been able to develop any feasible work arounds. Remote work is nearly impossible. All lab members are writing review articles.*  *No workarounds as most of my barriers involve home life. My workday is now whenever I can squeeze in the time/space.* |
